# Vectorial dynamics underpinning current and future tick-borne virus emergence in Europe

**DOI:** 10.1099/jgv.0.002041

**Published:** 2024-11-11

**Authors:** Marine J. Petit, Nicholas Johnson, Karen L. Mansfield

**Affiliations:** 1 Faculty of Health and Medical Sciences, University of Surrey, Guildford GU2 7XH, UK; 2 Animal and Plant Health Agency, Addlestone KT15 3NB, UK

**Keywords:** climate change, emerging viruses, tick-borne diseases, tick immunity, vector competency

## Abstract

Tick-borne diseases pose a growing threat to human and animal health in Europe, with tick-borne encephalitis virus (TBEV) and Crimean-Congo haemorrhagic fever virus (CCHFV), vectored by *Ixodes ricinus* and *Hyalomma marginatum*, respectively, emerging as primary public health concerns. The ability of ticks to transmit pathogens to multiple hosts and maintain infections across life stages makes them highly efficient vectors. However, many aspects of tick ecology and vectorial capacity remain understudied. This review examines key factors contributing to the vectorial competence of European ticks and their associated viruses. We first explore the influence of climate change on vector and disease ecology, using TBEV and CCHFV as case studies. We then analyse the role of the tick antiviral response in shaping vector competence. By integrating these elements, this review aims to enhance our understanding of tick-borne viral diseases and support the development of public health strategies, particularly through the One Health framework, to mitigate their impact in Europe.

## Introduction

Tick-borne viral diseases pose an escalating threat to human and animal health in Europe, with tick-borne encephalitis virus (TBEV) and Crimean-Congo haemorrhagic fever virus (CCHFV) in particular emerging as primary public health concerns [1,3]. These pathogens, along with localized viruses like louping-ill virus [4] and Uukuniemi virus (UUKV) [5], are transmitted by ticks and can cause severe neurological complications or fatal haemorrhagic fevers. The annual incidence of tick-borne encephalitis (TBE) has risen dramatically, with thousands of cases reported across central and eastern Europe [6]. Concurrently, Crimean-Congo haemorrhagic fever (CCHF) outbreaks with high fatality rates have expanded into new regions, including Spain and Portugal [7,8].

Several studies have associated climate change and global warming with the emergence and spread of vector-borne diseases [9,10]. Warmer temperatures and changes in precipitation patterns have extended the range and seasonality of ticks, thereby increasing the risk of human and animal exposure to tick-borne viral diseases across Europe [1,11]. Although temperature and humidity affect vector and virus biology, their relationship to the prevalence of infected ticks in Europe (0.1–5 %) [12,13] and their influence on tick vectorial capacity remain unclear.

**Fig. 1.**
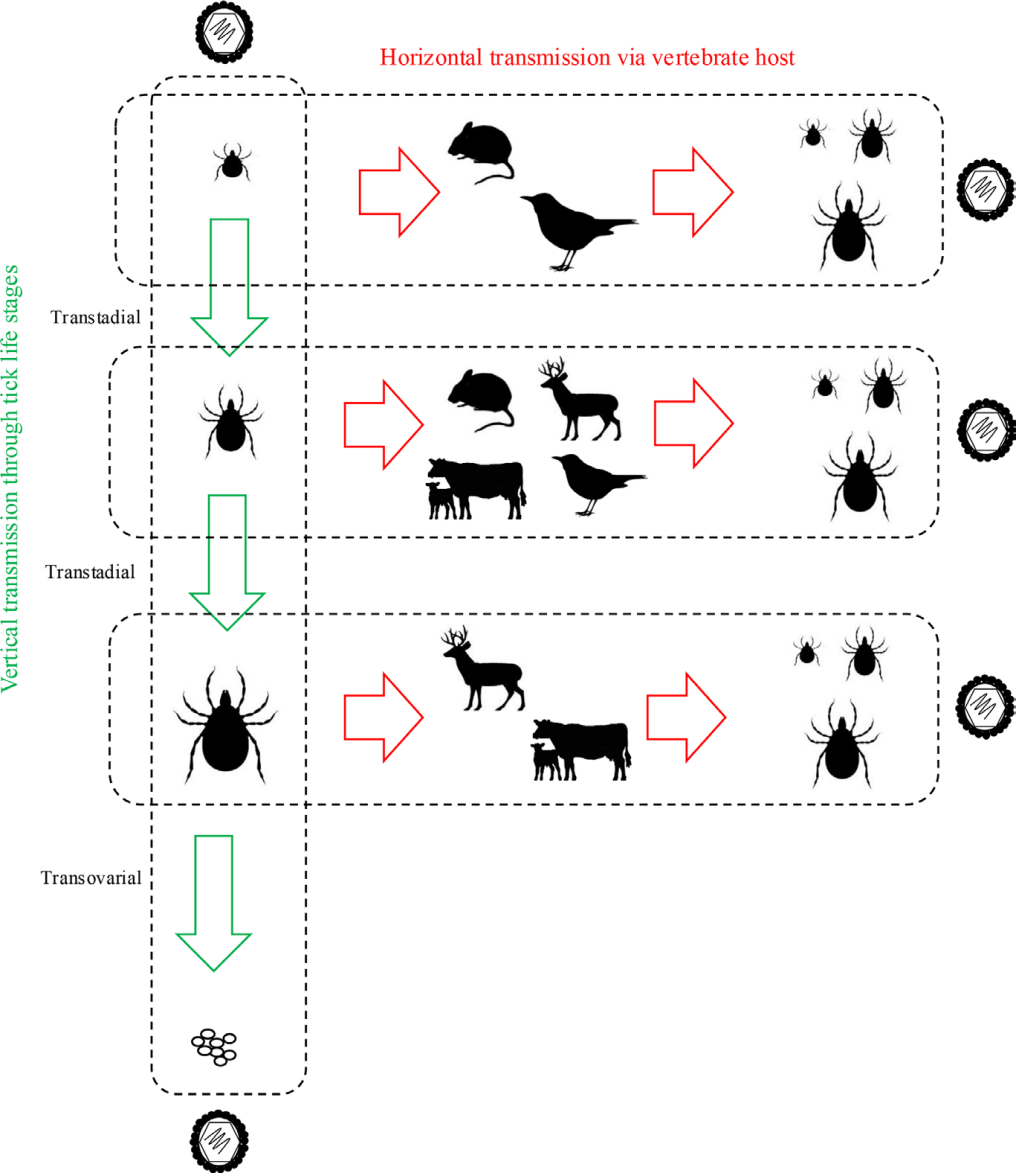
Transmission dynamics of TBEV between ticks and reservoir hosts. Vertical transmission through transstadial and transovarial transmission is shown in green. Horizontal transmission between the tick and vertebrate hosts is shown in red.

Many species of ticks in the families Ixodidae (hard ticks) and Argasidae (soft ticks) are associated with tick-borne virus transmission. The life cycle of hard ticks may involve one, two or three hosts, with nymphs and adult females feeding for prolonged periods (several days to weeks) [14]. In comparison, the life cycle of soft ticks includes several nymph stages, with nymphs and adults able to engorge up to ten times their body mass within a few minutes or hours [14]. Ticks transmit pathogens across multiple life stages, requiring viruses to persist through the moulting process and multiple blood meals, a characteristic distinct from other vectors such as mosquitoes. This aspect of tick biology shapes their vectorial capacity and creates complex epidemiological relationships with reservoir and spillover hosts (Fig. 1).

This review aims to provide a comprehensive analysis of the factors influencing tick vectorial capacity for viral diseases in Europe. We propose to examine European tick-borne viruses and their primary vectors, exploring predictions for tick population changes driven by environmental factors [15] and investigating the mechanisms underlying tick vectorial capacity, including the following: (1) tick-virus specificity, tick species diversities and distribution; (2) environmental influences, including climate variations, as well as change in reservoir host movement, density and distribution and (3) biological factors, specifically the tick antiviral immune responses that contribute to biological vector competence. By integrating these elements, we aim to deepen our understanding of tick-borne viral diseases and inform public health strategies to mitigate their impact in Europe, particularly through localized and continental One Health approaches that consider human, animal and environmental health [16].

## European tick-borne viruses

There are many tick-borne viruses circulating in Europe, detected in both ticks and wild mammalian species, and several have the potential to impact the health of humans, domestic animals and wildlife. Additionally, changes to climate, land use and human activities have facilitated changes to tick population dynamics, which in turn are impacting tick-borne disease transmission and facilitating the emergence of novel tick-borne viruses [17]. European tick-borne viruses with potential to cause severe disease in mammalian species include African swine fever virus (ASFV) (pigs and wild boar), louping-ill virus (cattle, sheep and red grouse) and louping-ill-like viruses such as Greek goat encephalitis virus, Spanish sheep encephalitis virus and Spanish goat encephalitis virus, while human pathogens with potentially fatal consequences include TBEV and CCHFV. A summary of key arthropod-borne viruses detected in European tick species that may impact animal and/or human health is reviewed by Hubalek and Rudolf [18] and detailed in Table 1. In terms of the burden of human disease, TBE is the most important tick-borne viral disease in Europe, caused by infection with TBEV following a tick bite or consumption of unpasteurized milk products [19]. The European sub-type of TBEV is detected widely across the European continent and is predominantly associated with *Ixodes ricinus* ticks [20]. At the borders of Europe, CCHFV is endemic in regions of the Balkans; however, in recent years, the virus has emerged in Europe to cause small numbers of human cases through a tick bite in Spain, Portugal and Bulgaria (*n*=17, 1 and 43 cases since 2013, respectively) [21], associated with ticks of the *Hyalomma *spp. genus [13]. However, the tick-borne virus of most significance to animal health in Europe is ASFV, and reports of disease in domestic pigs and wild boar continue to increase in several countries, including Bosnia and Herzegovina, Italy, Latvia and Poland [22]. Although the vast majority of tick-borne pathogens are transmitted by hard ticks in Europe, ASFV is one of the notable exceptions, as it is transmitted primarily by soft ticks of the *Ornithodoros *genus [23,24]. In Africa, the predominant tick vector for ASFV has been shown to be *O. moubata* [23,25], although the tick vectors in Europe are less clear. The soft tick *O. erraticus* was previously shown to be associated with an outbreak of African swine fever in domestic pigs on the Iberian Peninsula in 1957 [23,26]. The hard tick species *Dermacentor reticulatus* and *I. ricinus* may have the potential to act as ASFV vectors in Europe [24], although an assessment of tick-borne transmission of ASFV in Poland and the Baltic states concluded that European hard tick species would be unlikely to be involved in virus transmission [27], with other mechanisms of transmission more likely, such as direct pig-to-pig transmission or through environmental contamination with virus.

**Table 1.** European tick-borne viruses with impact on animal or human health

Virus	Genus	Disease impact (animal/human)	Tick species	Distribution in Europe	References
**Family: *Asfarviridae***					
ASFV	*Asfivirus*	Animal	*O. erraticus*	Widespread, including Sweden, Sardinia, Spain, Portugal, Germany, Italy, Latvia, Poland, Chechia, Greece, Bulgaria, Romania, Serbia, Montenegro, Bosnia and Herzegovina	[23,24]
**Family: *Bunyaviridae***					
Bhanja virus	*Bunyavirus*	Animal and human	*Haemophysalis punctata, H. sulcate* and *D. marginatus*	Italy, Croatia, Bulgaria and Slovakia	[137,138]
Avalon virus	*Nairovirus*	Human	*I. uriae*	France	[139]
Crimean-Congo haemorrhagic fever virus	*Nairovirus*	Human	Several including *D. marginatus, H. lusitarnicum, H. marginatum, I. ricinus, R. annulatus* and *R. sanguineus*	Bulgaria, France, Portugal and Spain	[140,142]
Soldado virus	*Nairovirus*	Animal (avian) and potentially human	*O. maritimus*	UK and France	[143,144]
St Abb’s Head virus	*Phlebovirus*	Animal (avian)	*I. uriae* and *I. rothchildi*	UK (Scotland)	[145]
**Family: *Flaviviridae***					
Greek goat encephalitis virus	*Flavivirus*	Animal	*I. ricinus*	Greece	[146,147]
Louping-ill virus	*Flavivirus*	Animal	*I. ricinus*	UK	[4,148]
Spanish goat encephalitis virus	*Flavivirus*	Animal	Potentially *Haemaphysalis* spp.	Spain	[149,150]
TBEV	*Flavivirus*	Human	*I. ricinus* and *I. persulcatus*	Widespread, including central Europe, Scandinavia and the UK	[19,20, 151]
Turkish sheep encephalitis virus	*Flavivirus*	Animal	Unspecified	Turkey	[152,153]
Tyuleniy virus	*Flavivirus*	Human	*I. uriae*	Kola Peninsula and Northern Russia (Europe)	[154]
West Nile virus*	*Flavivirus*	Animal and human	*H. marginatum*	Romania	[35]
Alongshan virus	*Jingmenvirus*	Human	*I. ricinus*	Finland, France, Germany and Switzerland	[28,29]
Jingmen tick virus	*Jingmenvirus*	Human	*R. bursa, H. marginatum, R. sanguineus* and *I. ricinus*	Romania, France and Poland	[17,30, 155]
**Family: *Orthomyxoviridae***					
Dhori virus	*Thogotovirus*	Human	*H. marginatum*	Portugal	[156]
Thogoto virus	*Thogotovirus*	Human	*R. bursa* and *R. sanguineus*	Sicily, Italy and Portugal	[157,158]
**Family: *Reoviridae***					
Eyach virus	*Coltivirus*	Animal and human	*I. ricinus* and *I. ventalloi*	France, Germany	[159,160]
Tribec virus	*Orbivirus*	Human	*I. ricinus, I. persulcatus* and *H. punctata*	Slovakia	[161,162]

* Isolated detections of mosquito-borne viruses in ticks.

There are also lesser-known tick-borne viruses circulating in Europe that do not cause serious disease in mammalian hosts or are under-reported, including Avalon virus, Bahig virus, Dhori virus, Eyach virus (EYAV), Thogoto virus, Tribec virus and Tyuleniy virus (reviewed in [18]; Table 1). Another such virus is Alongshan virus (ALSV), detected in *I. ricinus* in Finland, France, Germany and Switzerland [28,31]. ALSV can cause febrile illness in humans [32], with serological detection demonstrated in livestock [28,33]. There have also been isolated detections in European tick species of viruses more commonly associated with mosquitoes, including Sindbis virus (SINV, *alphavirus*) in *H. marginatum* ticks in Sicily, Italy [34], and West Nile virus (WNV, *flavivirus*) in a *H. marginatum* tick infesting a song thrush (*Turdus philomelos*) in Romania [35]. While ticks are not typically carriers of WNV, laboratory studies have reported some interesting results, where certain tick species, including *I. ricinus*, can become infected with WNV [36,37]. Although ticks are unlikely to transmit WNV effectively, they might serve as a potential reservoir, particularly as the virus was shown to persist as ticks progressed through their life stages, a process known as transstadial transmission [36,37]. However, a large-scale study assessing *H. marginatum* ticks infesting wild birds (*n*=14 824 birds) on the Mediterranean islands of Capri and Antikythera did not detect any WNV-positive ticks, suggesting that this tick species is unlikely to play a significant role in the spread of WNV from Africa to Europe [38]. Another mosquito-borne flavivirus, Usutu virus, has been detected in bird-associated *Ixodes* spp. ticks and shown to replicate in ticks [39]. There are also several viruses detected in European ticks, where there have been no reports of animal or human disease (Table S1, available in the online Supplementary Material). Many of these viruses are associated with *I. uriae* ticks, which parasitize seabirds, and include Puffin Island virus and Cape Wrath virus. Furthermore, several viruses that are normally associated with ticks and considered ‘tick-borne’ have also been detected in mosquitoes. These include UUKV (*phlebovirus*) and Zaliv Terpeniya virus (*phlebovirus*) [40,43] (Table S1).

In recent years, advances in molecular technology have facilitated metagenomic analysis of the entire tick virome, which has highlighted the true diversity of viruses harboured by ticks. Through integrated assessment of the entire tick microbial community, it is now possible to identify multiple pathogens that are closely associated with the diverse community of micro-organisms (including pathogens) harboured by ticks, and gain further insights into the influence of these pathogens on tick biology and pathogen persistence and transmission [44]. This methodology has identified an extensive range of viruses associated with ticks, some of which are insect-specific, and work in this field is rapidly expanding. This includes viruses associated with European *I. ricinus* such as Bronnoya virus, Chimay rhabdovirus, EYAV and several Norway viruses, including Norway nairovirus 1 and Norway phlebovirus 1 [45,49]. Viruses associated with European *D. reticulatus* include *D. reticulatus* pestivirus-like virus 1, * D. reticulatus* rhabdovirus 1 and *D. reticulatus* phlebovirus-like virus 1 [49]. While it remains unclear whether some of these viruses are transmissible, the investigation of the tick virome has revealed viruses from a diverse range of families, including *Flaviviridae, Bunyaviridae, Rhabdoviridae, Reoviridae* and *Phenuiviridae*.

## Tick vectors in Europe

**Fig. 2.**
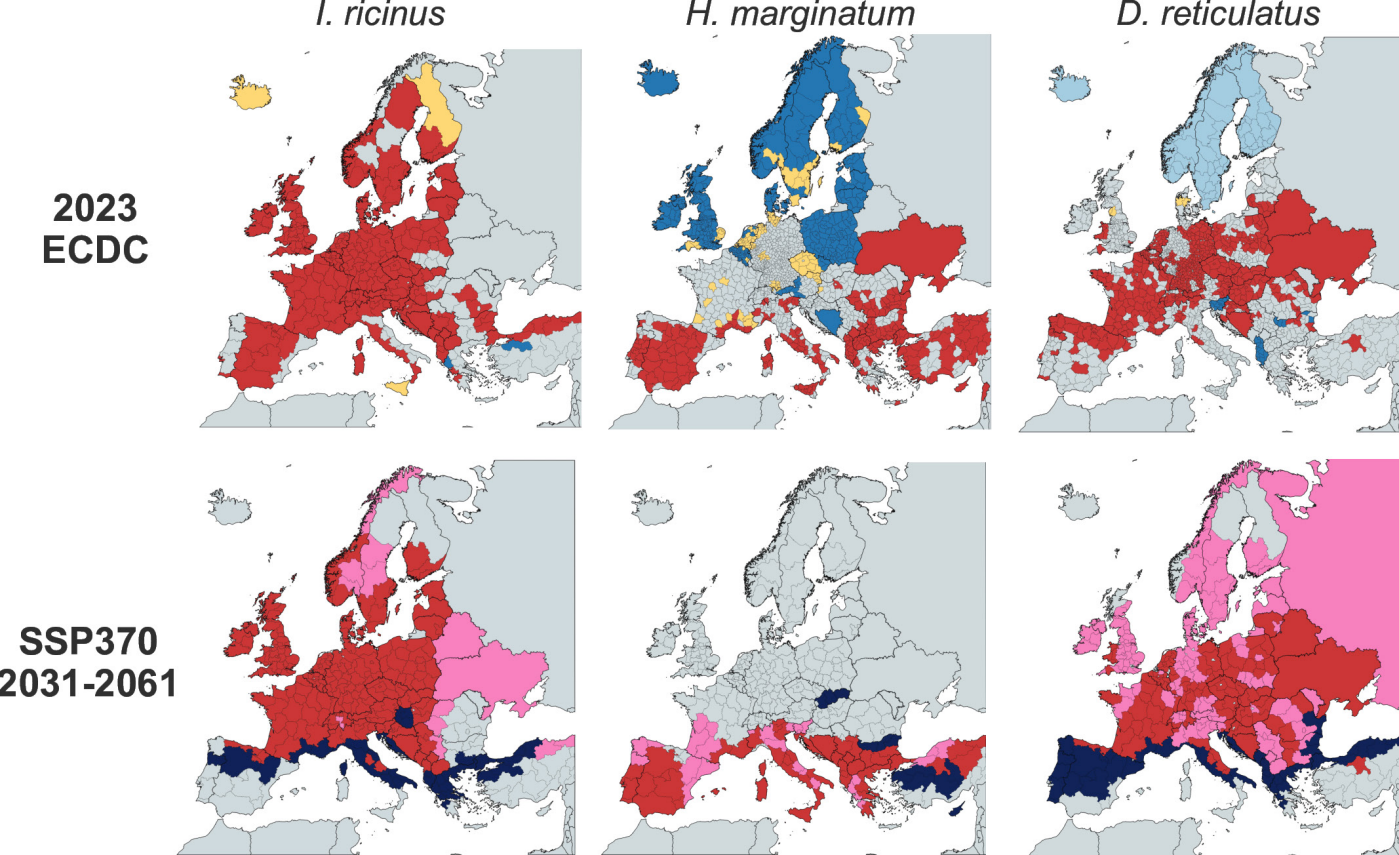
Current and future distribution of the main tick vector species in Europe. Distribution of *I. ricinus*, *H. marginatum* and *D. reticulatus* ticks in Europe, as of October 2023. Regions where the species is present are shown in red. Predicted introduction regions are in pink, while observed introduction regions are in yellow. Light blue indicates regions where the species is anticipated to be absent, blue where the species is observed absent, and dark blue represents regions with predicted absence. Adapted from the ECDC and European Food Safety Authority, 2023. SSP370 predictions for *I. ricinus* and *D. reticulatus* were adapted from Cunze *et al.* [163], and *H. marginatum* adapted from Hekimoglu *et al.* [61].

The predominant tick vectors of pathogens in Europe are the hard ticks *I. ricinus, I. persulcatus, H. marginatum* and *D. reticulatus*, and their geographical distributions are shown in Fig. 2 (European Centre for Disease Prevention and Control [ECDC], 2023). *I. ricinus* ticks are widely distributed throughout the European continent, which emphasizes their importance as a key vector species for multiple zoonotic tick-borne pathogens in Europe [50]. The geographical range of *I. ricinus* has expanded significantly in recent years, and this species is now detected in more northerly latitudes and at higher altitudes in Europe [51,52]. This expansion in range is associated with increases in tick abundance and extended periods of questing activity [53] due to multiple factors, including changes to climate, land use and wildlife distribution, along with socioeconomic factors increasing contact with humans [51,53, 54]. In comparison, *I. persulcatus* appears to be geographically restricted to northern and eastern regions of Europe, whilst *H. marginatum* appears restricted to southern and eastern countries in Europe. *D. reticulatus* appears widespread throughout Europe, with the exception of Scandinavia. Another ixodid tick species of importance is *I. uriae*, from which several seabird-associated viruses have been detected. Although some of these viruses may be pathogenic in avian hosts, generally, these viruses do not cause signs of disease in seabirds, and human infections are poorly understood [55]. In terms of soft tick species, *O. erraticus* is considered a potential vector of ASFV throughout Europe [23,26], whilst Soldado virus, Puffin Island Virus and Meaban virus have all been detected in *O. maritimus* [56,57].

## Changes to tick population dynamics – climate, land use and human activities

The diverse distribution and expanding ranges of European tick species highlight the complex and dynamic nature of tick-borne disease ecology. As these arthropod vectors continue to adapt to changing environments, their potential to transmit pathogens to new areas becomes increasingly significant. This evolving landscape of tick populations and their associated pathogens necessitates a deeper examination of the factors driving these changes, particularly in the context of global environmental shifts.

Changes in tick populations driven by factors such as climate change and land use modifications need to be characterized to understand their impact on tick-borne viral diseases in Europe. By associating surveillance datas and mathematical modelling, Lindgren was the first, in 1998, to predict that global warming and climate change will impact tick distributions in Europe [58]. Lindgrens’ key predictions have proven accurate, particularly the northward expansion of *I. ricinus* ticks and increased tick density in Europe due to milder winters and extended spring/autumn seasons. However, the study had several limitations, including its focus on Sweden, reliance on questionnaire data and limited consideration of factors beyond temperature.

Climate change projections, utilizing the WorldClim framework [59] and incorporating various shared socioeconomic pathways (SSPs), provide crucial insights into the future distribution of tick vectors. These models, developed within the Intergovernmental Panel on Climate Change framework, integrate complex socioeconomic factors such as economic growth, population dynamics, urbanization, technological progress and policy decisions to predict greenhouse gas emissions and subsequent climate outcomes. The SSP370 scenario, which assumes large carbon dioxide emissions and significant climate change, is particularly concerning. Unlike more sustainable pathways, SSP370 does not prioritize reforestation or rewilding efforts, focusing instead on continued land use intensification and environmental degradation. This scenario is widely used in tick prediction studies because it reflects a realistic medium-to-high emissions future where fragmented environmental policies and regional rivalry prevail. Under these conditions, projections for 2031–2061 suggest a dramatic shift in tick species distribution across Europe, with ticks expanding into northern regions that were previously unsuitable (Fig. 2). Notably, southern Europe may become inhospitable for *I. ricinus* and *D. reticulatus* [60], while simultaneously offering an ideal ecological niche for *H. marginatum*, the primary vector of CCHFV [61]. These projected changes align with observed trends, where elevated temperatures and humidity levels accelerate tick reproduction rates and prolong questing periods, fundamentally altering the seasonal dynamics of tick activity. However, those predictions remain limited due to the lack of long-term datasets on tick distribution, prevalence and abundance. In Europe, *Ixodes* sp. is detected at high altitudes [62,63], contrasting with the predicted pattern of migration towards northern latitudes [58]. While studies related to *I. ricinus* distribution in Norway and Sweden show a clear link between climate change and expansion, research in the UK presents a more complex picture. Studies in Scotland indicate that tick expansion is likely driven by an interplay of climate change, increasing host populations (especially deer) and other environmental factors [64,65].

**Fig. 3.**
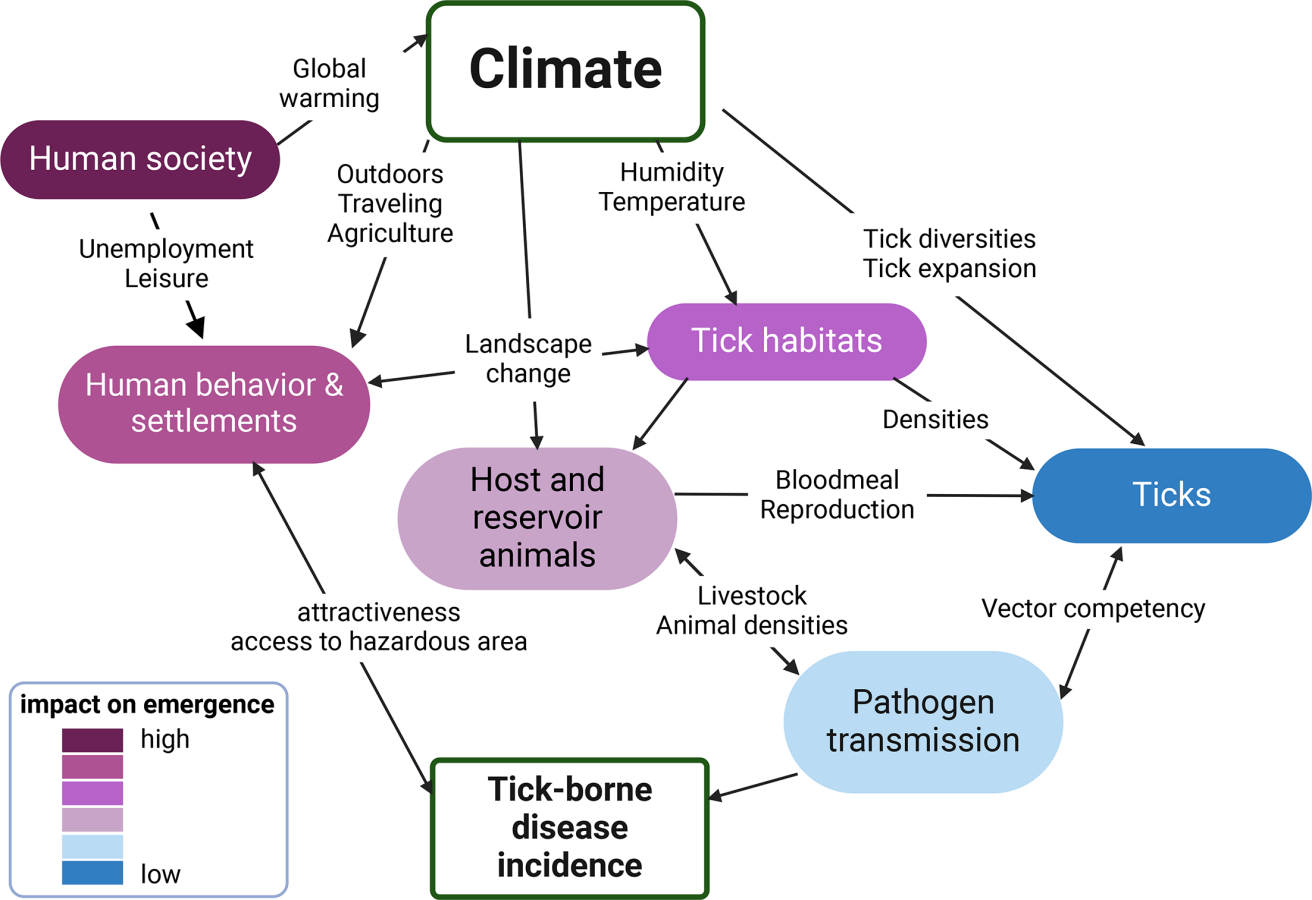
Representation of environmental, biological and human factors impacting tick-borne viral disease emergence. Adapted from Lindgren [58]. Each arrow represents identified drivers, facilitating different categories of impact, which are ranked from the most determinants to the least (adapted from [66]). Created in BioRender.

While we observe a clear expansion of tick species localization and densities, it remains difficult to connect vector changes to pathogen emergence. Modelling the spread and emergence of tick-borne viral diseases requires comprehensive modelling tools which include ecological, climatic and anthropogenic drivers (Fig. 3). A recent expert consortium ranked the drivers responsible for the increase of TBEV in Europe, revealing that human activity and behaviour are the primary factors, despite climate change influencing most other variables [66] (Fig. 3) . Landscape changes, including fragmentation, reforestation, management of unimproved pastures and transition areas (e.g. zones where two different types of land use or ecosystems meet or gradually change into one another) [67], along with climate-related factors such as temperature and humidity, significantly impact tick survival and disease dynamics [68]. These alterations affect the habitats, movement and density of wildlife hosts, consequently influencing the presence of reservoirs, vectors and viruses [69,70]. Moreover, these changes may increase human exposure to infected ticks by enhancing the attractiveness and accessibility of high-risk areas. Microclimatic temperatures play a crucial role in viral replication within vectors, affecting both the extrinsic incubation period and blood meal digestion duration [71,72]. The abundance and distribution of wildlife reservoirs, particularly rodents and small mammals, significantly impact pathogen spread, with higher densities often associated with increased viraemia and transmission rates [73,74]. Similarly, the presence and density of competent tick species directly influence disease transmission risk. While these factors provide valuable insights into the dynamics of tick-borne viral diseases, further data are needed to refine predictive models and improve their accuracy, as demonstrated by the recent trends in TBEV and CCHFV geographical emergence in Europe.

## Geographical expansion of tick-borne pathogens in Europe

### Continued European expansion of TBEV

The essential element for the proliferation of the TBEV primary vector, *I. ricinus,* is humidity, with dry weather causing a rise in saturation deficit and a decrease in nymphal questing activity [68]. Conversely, rising global temperatures are thought to contribute to the growth of *I. ricinus* populations by extending the tick questing season into winter and improving the winter survival of hosts, especially deer, and of the tick itself. However, global warming might increase the aridity of some *Ixodes-*abundant areas, potentially mitigating this effect. Interestingly, the dissemination of TBEV in Europe was predicted in the 2000s to be restricted to high latitudes and low altitudes, based on mathematical modelling of global warming’s impact [58]. This modelling considered factors such as enhanced conditions for natural transmission cycles, modified human behaviour resulting in increased tick exposure and altered agricultural methods, which increased TBEV food-borne transmission through increased raw milk intake [75]. Contrary to these predictions, recent studies have shown the presence of TBEV in the Netherlands, Belgium and the UK, indicating a westward spread of TBEV. Additionally, there are more cases reported in Central Asian countries [76]. The incidence of human cases in 2020 reached 3817 in 24 countries of the EU/EEA, showing a seasonal pattern and an incidence 50% more than the 2016–2018 baseline [77]. The most important driver of TBEV expansion is human behaviour and activities, leading to increased contact with the tick vector in high-risk areas with high TBEV prevalence [66]. This highlights the need for integrating mathematical modelling with strong surveillance and public health strategies to accurately predict and mitigate the spread of tick-borne diseases in Europe.

### Emergence and European expansion of CCHFV

CCHFV is expanding its geographical range in Europe, a trend closely associated with vector climate change. The original distribution of CCHFV included Africa, Asia and the Middle East; however, in recent years, the virus has emerged in Southern and Eastern Europe, with cases reported in Spain, Turkey, the Balkan countries and Greece [78,80]. This expansion is primarily attributed to the northward migration of *Hyalomma* tick species, the principal vector of CCHFV [81]. Rising temperatures and altered precipitation patterns create favourable conditions for tick survival and proliferation, facilitating this geographical shift. There is also increased awareness of CCHF and resulting surveillance within tick populations and natural hosts [82,83].

Predictive models incorporating temperature data, *Hyalomma* spp. habitat suitability and host distribution predict CCHF’s continued expansion into Central and Northern Europe as climate change persists [61]. These models, including ecological niche modelling and climate envelope models, consider factors such as temperature, humidity, vegetation cover and animal host movements to predict *Hyalomma* (Fig. 2) and CCHFV distribution across continental Europe, with a medium risk of CCHFV introduction for France, Italy and Germany [82]. However, the link between *Hyalomma* spp. presence and CCHFV emergence remains unclear. For instance, cases of CCHFV detection in Greece were associated with *Rhipicephalus* spp. ticks [83], demonstrating that CCHFV is vectored by multiple tick species. Such field observations are invaluable for refining current models. They provide valuable insights into the potential future distribution of CCHFV, informing public health strategies and surveillance efforts.

## Ticks as an efficient vector for viruses

Ticks successfully establish viral infections by ingesting pathogens during a haematophagous meal from an infected host. Post-ingestion, the virus traverses the midgut barriers and disseminates to critical organs such as the salivary glands, enabling transmission to a new host during subsequent blood meals [84]. Throughout the tick lifecycle, the virus must adeptly evade the tick’s innate immune defences, a crucial aspect for sustaining persistent infections that promote viral proliferation while safeguarding vector survival [85,86]. Unlike vertebrates, arthropods, including ticks, primarily depend on their innate immune defences to differentiate between self and non-self elements [87,88], deploying antiviral effectors to neutralize infectious viral particles [89,90]. The haematophagous nature of ticks exposes them to a broad array of pathogens from diverse vertebrate hosts [91,92], necessitating robust innate immune responses and cell-mediated mechanisms [93]. For the scope of this review, we will focus specifically on the innate immune response in ticks, exploring the mechanisms by which ticks detect and control viral replication within infected cells and tissues.

### RNA interference: primary antiviral defence of ticks

The small interfering RNA (siRNA) pathway is a major antiviral defence mechanism in arthropod vector cells, selectively targeting and destroying viral RNA. Most arboviruses, along with other viral families, produce double-stranded RNA (dsRNA), either as replication intermediates or genome components, which constitutes a key pathogen-associated molecular pattern (PAMP) recognized by the host immune system [94]. dsRNA is recognized by the RNA interference (RNAi) machinery, which processes it into 21–22 base pair siRNAs. These are then incorporated into the RNA-induced silencing complex (RISC), which facilitates the targeted degradation of complementary viral RNAs [95]. Genomic and comparative genomic analyses in *Ixodes* ticks have confirmed the conservation of key RNAi proteins, including RNAi effectors Dicer-2, Argonaute (Ago) 2, miRNAs Dicer-1 and Ago-1 and the piwi-interacting RNAs protein Aubergine [96,98]. Observed gene expansion of the Ago2 protein, 11 and 15 paralogs were identified in *I. persulcatus* and *Hyalomma* ticks, respectively, demonstrating a specialization of the tick siRNAs. Similarly, the absence of Vago [97], an siRNA effector supporting and amplifying RNAi antiviral action, in tick genome suggests a difference between the siRNA pathway in ticks and other arthropod taxa.

These structural variations of the tick siRNA machinery underscore the need to characterize the role played by siRNA in tick antiviral defence. siRNAs were produced in *I. scapularis* and *I. ricinus* cells infected with tick-borne flaviviruses [99,100] and efficiently decreased virion production, suggesting antiviral activity. In *I. scapularis* cells, the AGO2 proteins, Ago-30 and Ago-16, appear to mediate antiviral activity [99], confirmed by the identification of antiflavivirus siRNA in the whole tick [101]. Further characterization of the antiviral role of siRNA in *I. ricinus* cells infected with Langat virus dismisses the role of the conserved Tudor-SN protein in the antiviral response, indicating an alternative role in regulating gene expression through the activity of endogenous siRNAs [100]. In *Hyalomma* spp., siRNA activity restricts viral replication within two different viral families, specifically Hazara virus (*Bunyavirales* [102]) and SINV (*Alphavirales* [103]). siRNA therefore appears to constitute a pan-viral defence system in tick vector cells, and while complete viral clearance by siRNA was never achieved, this only suggests that ticks, like other arthropods, combine immune pathways to efficiently control viral replication and infection [104].

### Antiviral detection and response in tick cells

**Fig. 4.**
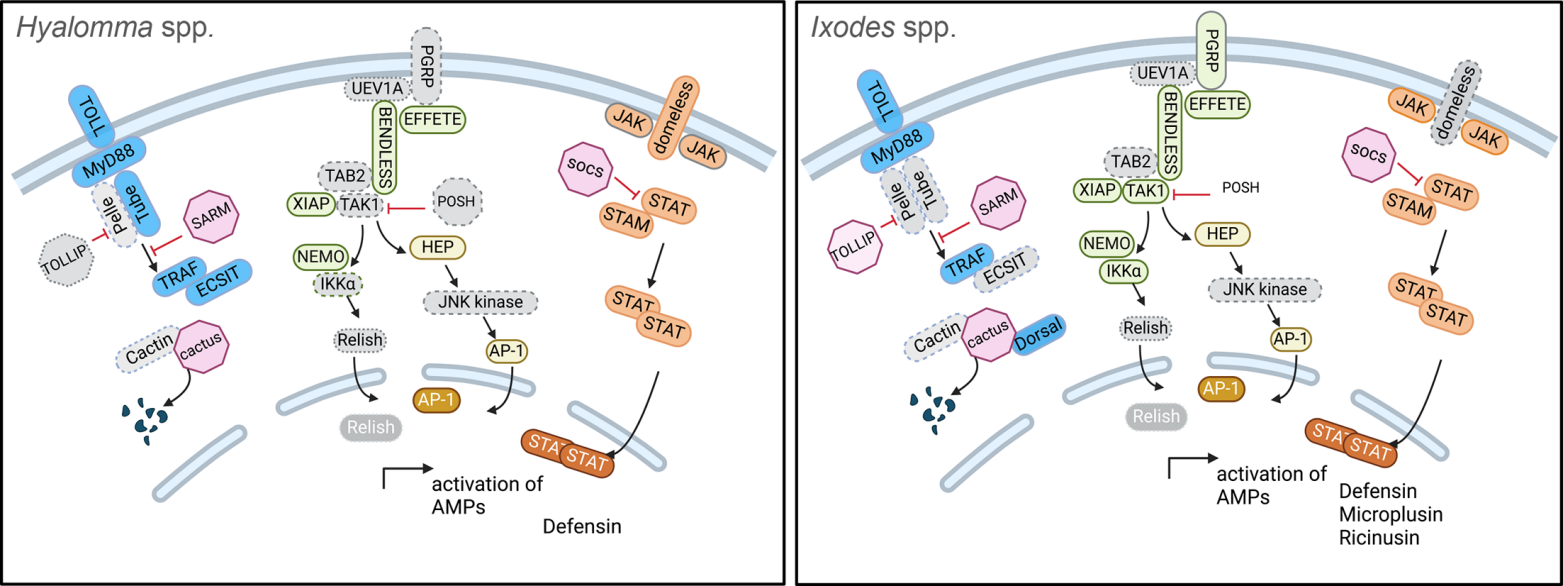
Innate immune pathways of the main European tick vectors, *Ixodes* spp. and *Hyalomma* spp. Schematic representation of the JAK-STAT (orange), Imd (green), Toll (blue) and c-Jun N-terminal kinase (yellow) pathways adapted from [125] for *Hyalomma* spp. and for *Ixodes* spp. [92,93, 103, 126]. Plain coloured proteins are known factors, dashed grey proteins represent non-identified proteins. Created in BioRender.

The detection of PAMPs such as glycoproteins and nucleic acids triggers signalling cascades that facilitate the translocation of transcription factors to the nucleus, which is a crucial step for the induction of antiviral gene expression [105]. Arthropod core immune pathways, such as the Janus kinase and signal transducer and activator of transcription proteins (JAK-STAT) pathway, along with the immune deficiency (Imd) and Toll pathways (Fig. 4), have all been identified in all tick vector species that have been investigated [97]. However, the different levels of genome quality and annotation make it difficult to identify each and all immune cascade effectors, limiting our understanding of their significance in antiviral responses.

In the tick antiviral responses, the Toll pathway emerges as a predominant mechanism in both infected cells and tissues [89,90, 106]. The Toll pathway is ubiquitously present across European tick vector species, including *I. ricinus* and *Hyalomma* spp., and exhibits a high degree of conservation [96,97, 107]. The architectural integrity of the Toll pathway in ticks mirrors that observed in insects, with most components being preserved; however, notable deviations exist (Fig. 4, blue pathway) [96,108]. For instance, *I. persulcatus* hosts 11 Toll receptors, *I. scapularis* 10, whereas *Hyalomma* spp. possess only 9 [97]. Additionally, while the effector Spätzle is conserved in *Ixodes*, it is either absent or remains unidentified in *Hyalomma*, underscoring interspecies variability [97]. Another difference observed when comparing *Drosophila melanogaster* and tick Toll pathway is the absence of the Dif factors, which have been shown to be essential for the expression of antimicrobial peptides (AMPs) in insects, notably Defensin expression [109]. This suggests an alternative mechanism for the expression of AMPs in *Ixodes* spp. The antiviral activity of the Toll pathway in ticks has been relatively underexplored, with limited studies addressing its role. Notably, Mansfield *et al.* [89] identified a flavivirus-specific upregulation of a single Toll-like protein transcript (ISCW022740), following Langat and louping-ill virus infection in *I. ricinus* cells. However, only louping-ill infection led to the production of the AMP ISCW014204, which includes a beta-transducin motif [89] and is characterized as an NF-kB antiviral in crustaceans [110]. These findings imply that the Toll pathway induces a selective antiflaviviral activity in infected tick cells [89,111]. While the Toll pathway has been identified as antiviral in the gut tissues of mosquitoes and *Drosophila* [112,113], further evidence is required to elucidate its role in the antiviral response of tick midgut and haemolymph cells. The absence of Toll pathway activation in salivary glands indicates that it does not play a role in viral transmission to new hosts [114].

In addition to the Toll pathway, tick genome encoded most of the effectors of the Imd pathway, which is another tick innate immune pathway implicated in the antimicrobial response [115]. Notably, the conservation of the Imd pathway among arthropod vectors exhibits considerable variability, as illustrated by the absence of the Imd receptor and the Drosophila Fas-associated death domain effector in all sequenced tick genomes (Fig. 4). Despite the absence of these pivotal components, the Imd pathway retains functionality within ticks and tick-derived cell lines, as primarily evidenced in *I. scapularis* [116]. O’Neal *et al.* [115] demonstrated that in *Borrelia burgdorferi*-infected *I. scapularis* ticks, the scavenger receptor Croquemort (Crq) plays a crucial role in recognizing lipid patterns, compensating for the canonical Imd receptor peptidoglycan-recognition protein (PGRP), which relies on peptidoglycan recognition. Contrastingly, in *Hyalomma* spp., the Imd pathway exhibits further deficiencies, lacking additional critical effectors such as the β−1,3-glucan recognition protein, which are integral to insect and tick defence mechanisms [117]. The advancement in genome annotation and quality is therefore needed to elucidate the signalling cascades in *Hyalomma*, thereby enriching our comprehension of antiviral responses within these species. While numerous studies have delineated the antiviral capacity of the Imd pathway in Drosophila and mosquitoes, investigations into tick cell infections with flaviviruses, alphaviruses and bunyaviruses have yet to affirm the antiviral role of the Imd pathway. This discrepancy may be attributed to the absence of Imd receptor which recognizes viral PAMPs.

Closely associated to the Imd pathway, c-Jun N-terminal kinase (JNK) pathway is associated with stress responses and bacterial immunity in *Ixodes* sp. (Fig. 4, yellow pathway). A recent study on TBEV-*I. ricinus* protein–protein interactions has identified an enrichment of proteins belonging to the response to cytokine category [106], specifically the JunD factor which acts as an activator of the JNK pathway transcription factor AP-1. In the context of TBEV infection, JunD interacts with the viral protein NS5, potentially influencing the transcription of AP-1-regulated genes [106]. It remains unclear if the JunD–NS5 interaction may either enhance a viral-beneficial transcriptional response or suppress the host’s antiviral response. Finally, the last arthropod core immune pathway described in all tick vector species is the Jak-STAT pathway (Fig. 4, orange pathway). JAK/STAT is not part of the humoral innate response in arthropods but does have a role in immunity through crosstalk with IMD and Toll signalling in insects [118]. Comparative genomic analysis demonstrated the conservation of the JAK/STAT pathway between ticks and *Drosophila* [96], apart from the unpaired protein [119,120]. Despite the robust conservation of the STAT pathway and observations of TBEV NS5-mediated STAT antagonism in human cells [121], this suggests potential conservation of this interaction and its antiviral role in ticks, and recent protein–protein interaction studies in tick cells indicate otherwise [106]. To date, no antiviral functions have been definitively attributed to the tick JAK-STAT pathway. This gap in knowledge underscores the limitations imposed by current study designs. Future research employing targeted dsRNA silencing of JAK-STAT effectors could elucidate whether the antiviral function of the JAK-STAT pathway is conserved across vertebrate hosts and tick vectors. The activation of immune signalling pathways results in the expression of AMPs, a conserved class of immune peptides. While ticks, including *Ixodes* sp. and *Hyalomma* sp., produce various AMPs, including defensins, lysozymes, lectins, proteases and protease inhibitors, only defensins and defensin-like AMPs have been extensively assessed for their antiviral properties [117,122]. Defensins are typically 18–45 amino acids long and characterized by a β-sheet structure stabilized by three or four disulphide bonds. They are the most studied tick AMPs and have demonstrated antiviral activity against various RNA and DNA vector-borne viruses [85,87]. For instance, synthetic defensin-like peptides from *Haemaphysalis longicornis* exhibited virucidal activity against the tick-borne flavivirus Langat virus *in vitro* [123]. Interestingly, new research has highlighted the potential of tick-derived AMPs in controlling ASFV infection. OPTX-1, a defensin-like peptide toxin produced from *O. papillipes*, was discovered to be a competitive inhibitor of ASFV pS273R protease, effectively reducing ASFV replication [124]. Notably, hard tick-derived defensins exhibit considerably more specific inhibitory effects on the pS273R protease, indicating that defensins may play a role in vector competence [124]. These findings highlight the complex relationship between tick AMPs and viral infections, indicating that while some AMPs may contribute to viral control, their roles *in vivo* may be more nuanced and diverse than initially thought. Further research is needed to fully elucidate the biological roles of native AMPs in the tick-virus interface. To advance our understanding of AMP action, it is essential to clearly elucidate the activation of signalling cascades and their connections to the transcription factors AP-1, Dorsal and STAT, which are involved in the regulation of AMP expression *in vitro* and *in vivo*.

### Cellular immunity within the tick

Beyond intra-cellular defences, ticks also depend on cellular immunity to control and eliminate viruses. This response is primarily mediated by haemocytes, which consist of four distinct types of cells present within the tick’s haemolymph [125]. Both hard ticks infected with the *Orthomyxoviridae* Thogotovirus or soft tick infected with ASFV showed a high virus titre and the presence of viral antigens in their haemolymph [125,126]. Haemocytes employ three main mechanisms: phagocytosis, encapsulation and nodule formation. Phagocytosis, regulated by a complement-like system, involves haemocytes recognizing and engulfing viral particles through pattern recognition receptors (PRRs). In *I. ricinus*, C3-like molecules (IrC3-1, IrC3-2, IrC3-3) are involved in this process [127]; however, no studies have yet demonstrated the role of phagocytosis in antiviral defences for European ticks. Encapsulation and nodulation are both extracellular trap mechanisms. Encapsidation occurs when haemocytes surround groups of viruses, while nodulation involves haemocyte aggregation around a cluster of viruses. Nodule formation is particularly important in ticks as it helps contain and isolate infections, preventing their spread within the body of the tick. This process is especially effective against larger numbers of pathogens that cannot be efficiently eliminated through phagocytosis alone. Finally, haemolymph also facilitates the circulation of antiviral peptides and reactive oxygen species throughout the tick vector, enhancing viral control in various tick organs. Despite these insights, understanding of the tick cellular immunity remains scarce due to limited knowledge of the heterogeneity of haemocyte populations and the difficulty in isolating and characterizing specific cell types. To overcome these limitations and gain a more comprehensive understanding of tick immune responses, single-cell RNA sequencing of haemocytes could be employed [128]. This approach would allow for the identification of distinct subpopulations, their gene expression profiles and their specific roles in antiviral immunity.

## Future perspectives

The interconnections between human, animal, climate and vector activities are crucial for the dissemination and maintenance of tick-borne viruses in the environment. Addressing this multi-factorial challenge requires a multi-faceted approach that integrates vector and host knowledge, molecular and population-level interaction characterization and environmental studies. One Health strategies offer a comprehensive framework for such efforts as they are integrated, collaborative approaches that address health risks at the interface of humans, animals and ecosystems to achieve optimal health outcomes [16,129]. By employing One Health strategies, we can develop more effective and sustainable solutions to combat tick-borne diseases [130].

While the growth of a citizen science culture and awareness about the impact of climate and biodiversity on viral emergence has improved recently [131], the tick-borne disease field remains neglected. Therefore, significant efforts to develop novel and robust surveillance tools (tick species and disease monitoring), diagnoses and knowledge on tick–host–virus interactions must be investigated. It is also essential that local and national public health institutions, as well as environmental agencies, participate in and support One Health’s zoonotic disease control and prevention efforts. At the European level, there has been a significant effort in surveillance, epidemiology and predictive modelling, primarily coordinated by the ECDC [77,132], along with individual countries such as Italy that are at risk for tick-borne virus emergence [133,134]. Recently identified as a medium-risk area for CCHFV emergence [82], Italy has developed extensive surveillance programmes for cattle, ticks and migratory birds [135,136]. Although no human cases have been detected so far in Italy, continuous efforts are necessary as no treatment or vaccine is currently available for CCHFV in humans or animals.

## Conclusions

Our review highlights significant progress in understanding tick–virus interactions over the past decades. However, to develop novel strategies to control and prevent the spread of existing and emerging viruses, research must be strengthened in three strategic areas:

1. Superior annotation of tick genomes: Crucial for identifying innate immune homologues, genome annotations provide insights into the molecular interactions between ticks and viruses and enhance our understanding of tick-borne virus transmission.
2. AMP discovery and study: AMPs present a promising avenue for therapeutic development, showing potential in controlling virus replication within tick vectors.
3. Impact of climate change on virus transmission: Global warming significantly impacts the prevalence and distribution of virus-infected ticks, altering the risk of virus transmission to humans and animals. Understanding these dynamics is essential for developing effective surveillance, prevention and control strategies.

## Supplementary material

10.1099/jgv.0.002041 Uncited Table S1.

## Abbreviations

AMP: antimicrobial peptide
AMP: antimicrobial peptide
ASFV: African swine fever virus
CCHF: Crimean-Congo haemorrhagic fever
CCHFV: Crimean-Congo haemorrhagic fever virus
dsRNA: double-stranded RNA
ECDC: European Centre for Disease Prevention and Control
EU/EEA: European Union/European Economic Area
EYAV: Eyach virus
Imd: immune deficiency
Jak-STAT: Janus kinase and signal transducer and activator of transcription
JNK: c-Jun N-terminal kinase
PPR: Pattern Recognition Receptors
RNAi: RNA interference
SINV: Sindbis virus
siRNA: small interfering RNA
SSP: shared socioeconomic pathway
TBEV: tick-borne encephalitis virus
UUKV: Uukuniemi virus
WNV: West Nile virus
