# Supplementary material for: Vectorial dynamics underpinning current and future tick-borne virus emergence in Europe
Source: J Gen Virol. 2024 Nov 11;105(11):002041. doi: 10.1099/jgv.0.002041 (PMC12455053; doi:10.1099/jgv.0.002041)
Supplement: Uncited Table S1. [file jgv-105-02041-s001.pdf]

Virus	Genus	Tick species	Distribution in Europe	References
**Family: *Bunyaviridae***				
Clo Mor virus (CMV)	*Nairovirus*	*Ixodes uriae*	UK	(164)
Puffin Island virus (PIV)	*Nairovirus*	*O. maritimus, Ixodes uriae*	UK	(82)
Bahig virus (BAHV)	*Orthobunyavirus*	*Hyalomma marginatum*	Italy	(165)
Matruh virus (MTRV)	*Orthobunyavirus*	*Hyalomma marginatum*	Italy	(166)
Grand Arbaud virus (GAV)	*Phlebovirus*	*Argas reflexus*	France	(167)
Ponteves virus (PTVV)	*Phlebovirus*	*Argas reflexus*	France	(167)
Uukuniemi virus (UUKV)*	*Phlebovirus*	*Ixodes ricinus, Ixodes persulcatus*	Finland, Chechia	(66, 168)
Zaliv Terpeniya virus (ZTV)*	*Phlebovirus*	*Ixodes uraie*	France, Northern Russia (European, Murmansk)	(68, 69)
**Family: *Flaviviridae***				
Meaban virus (MEAV)	*Flavivirus*	*O. maritimus*	France	(83)
**Family: *Reoviridae***				
Bauline virus (BAUV)	Orbivirus	*Ixodes uriae*	Norway	(169)
Cape Wrath virus (CWV)	Orbivirus	*Ixodes uriae*	UK	(170)
Mykines virus (MYKV)	*Orbivirus*	*Ixodes uriae*	Faroe Islands	(171)
Okhotskiy virus (OKHV)	*Orbivirus*	*Ixodes uriae*	Northern Russia (European, Murmansk)	(172)
Tindholmur virus (TDMV)	*Orbivirus*	*Ixodes uriae*	Faroe Islands	(171)

Supplementary Table S1: European tick-borne viruses with no known or reported impact on animal and human health.

**Isolated detections of tick-borne viruses in mosquitoes.*
